# Effect of suppression of otoacoustic emissions in individuals with and without central auditory processing disorder: a systematic review

**DOI:** 10.1016/j.bjorl.2024.101485

**Published:** 2024-08-09

**Authors:** Jéssica Dayane da Silva, Mariana de Carvalho Leal Gouveia, Laís Cristine Delgado da Hora, Leonardo Gleygson Ângelo Venancio, Lilian Ferreira Muniz

**Affiliations:** a Universidade Federal de Pernambuco (UFPE), Programa de Pós-Graduação em Saúde da Comunicação Humana, Recife, PE, Brazil; b Universidade Federal de Pernambuco (UFPE), Departamento de Cirurgia, Recife, PE, Brazil; c Universidade Federal de Pernambuco (UFPE), Mestrado em Saúde da Comunicação Humana, Recife, PE, Brazil; d Universidade Federal de Pernambuco (UFPE), Programa de Pós-Graduação em Cirurgia, Recife, PE, Brazil; e Universidade Federal de Pernambuco (UFPE), Departamento de Fonoaudiologia, Recife, PE, Brazil

**Keywords:** Auditory perception, Auditory perception disorders, Efferent pathway, Otoacoustic emissions, Suppression of otoacoustic emissions

## Abstract

**Objective:**

To investigate, through a systematic review of the literature, whether there are differences in the results of otoacoustic emissions suppression tests in individuals with and without central auditory processing disorder.

**Methods:**

Searches were carried out in the scientific databases Latin American and Caribbean Health Sciences Literature, PubMed, Scientific Electronic Library Online, Web of Science, Scopus, Science Direct and Cochrane, as well as the databases of gray literature British Library, OpenGrey.eu and Object View and Interaction Design. A systematic review of the literature was carried out, with the descriptors otoacoustic emissions and auditory perceptual disorders and their synonyms, combined by the Boolean operators AND and OR. The reading of the studies was done by peers independently and in case of disagreement in the inclusion of studies, a third researcher was consulted. Original cross-sectional articles with a comparison group that suppressed transient evoked otoacoustic emissions in individuals with and without central auditory processing disorder were included.

**Results:**

Seven studies that evaluated children aged between 7 and 14 years old were included, with methodological variability in the performance and analysis of the exam, the contralateral noise was the most used to elicit suppression. In only three studies did the group with central auditory processing disorder show lower suppression values, however the meta-analysis shows significant differences between the groups, with lower suppression values in the study groups.

**Conclusion:**

This study points the need for a protocol that standardizes the measurement of OAE suppression and its findings, aiming to reduce the inclusion of results unrelated to the olivocochlear system.

## Highlights

• Different results of TEOAE suppression tests in individuals with and without CAPD.
• It is suggested to standardize the measurement and analysis parameters of the exam.
• Studies that modify parameters and reduce responses of different nature than MOC.

## Introduction

This article has a level 4 of evidence, according to verification by the Oxford Center for Evidence-Based Medicine 2011, since the studies included are observational studies with a comparative group, which brings limitations regarding the quality of the findings of this review.

The Central Auditory Processing (CAP) refers to mechanisms and processes of the central auditory nervous system used to interpret ambient sounds.^1^ In the adult population, the complaints of alterations in the CAP are mainly related to the difficulties in communicational, social, language learning situations and the perception of supra-segmental characteristics of speech, which can compromise social and professional performance.^2^

Its evaluation occur behaviorally, through a battery of standardized tests, in an acoustic booth and although this form of evaluation is scientifically well established, such tests are not part of the audiological battery in the clinical routine and are requested by health professionals, generally, when patients have behavioral signs and complaints that indicate difficulty in understanding acoustic signals with normal peripheral auditory acuity.^3^

Difficulty in intelligible speech in noisy environments is one of the main complaints of individuals with CAPD. Such difficulty may be related, more specifically, to changes in the efferent auditory pathway and may indicate central disorders in the encoding of received acoustic stimuli and impairment of auditory biofeedback.^4^

Understanding that the mechanisms that favor the encoding of speech signals are not restricted to cortical activity helps to identify other structures and auditory pathways that may also be compromised in patients with hearing difficulties in noisy environments.^4^, ^5^, ^6^, ^7^

The suppression test of Transient Evoked Otoacoustic Emissions (TEOAE) has been pointed out as a promising instrument to verify the functionality of the efferent auditory system, considering the ease and speed of the performance and evaluation of this method.^8^, ^9^, ^10^ TEOAE suppression has been performed in patients with Central Auditory Processing Disorder (CAPD) or in suspected cases.

The suppression of Otoacoustic Emissions (OAEs) may be associated with CAPD for several reasons. Firstly, the lack of suppression of OAEs may indicate a disconnection between peripheral and central auditory pathways, suggesting a failure in the efficient transmission of auditory information to higher brain areas. Additionally, the modulation of OAEs by neural mechanisms, such as the Medial Olivocochlear System (MOCB), is crucial for regulating cochlear sensitivity, and dysfunction in this system may contribute to both the absence of OAE suppression and difficulties in central auditory processing, such as the integration of auditory information and temporal and spatial discrimination.^11^

Some aspects can interfere with the measurement of OAE suppression, one of which is the Middle Ear Muscle Reflex (MEMR). When the reflex is present, especially at high levels, it can cause a false reduction in OAEs, leading to a mistaken interpretation of suppression absence. Additionally, a low Signal-to-Noise Ratio (SNR) can compromise the accuracy of OAE suppression assessment. Noisy environments or measurement equipment with low sensitivity can hinder the detection of OAEs, resulting in inaccurate outcomes.^12^, ^13^

Therefore, when interpreting the results of OAE suppression, it is important to consider these limitations and conduct the assessment in controlled conditions, minimizing the influence of the middle ear muscle reflex, and ensuring an adequate signal-to-noise ratio for a precise interpretation of the results. Without correctly capturing the MOCB responses, it is not possible to observe their effects on the CAP.

Thus, alterations in the efferent auditory pathway may be involved in a CAPD of sound information and considering that the analysis of TEOAE suppression is a tool that can be quickly applied. The need to investigate through a systematic literature review is highlighted whether there are differences in the results of TEOAE suppression tests in individuals with and without CAPD, as well as verifying whether changes in stimulation parameters may make it difficult to observe these results.

## Methods

The systematic review of the literature was registered and approved on the PROSPERO platform under registration number CRD42020191446.

To carry out this work, the following guiding question was used: Does the TEOAE suppression effect have different behavior in individuals with and without CAPD?

The POT strategy used was defined as: Population (P) ‒ Individuals with and without CAPD; Outcome (O): TEOAE suppression effect in individuals with and without CAPD; Type of study (T): Cross-sectional study.

### Search strategy

The databases Latin American and Caribbean Literature in Health Sciences (Lilacs), PubMed and Scientific Electronic Library Online (SciELO), Web of Science, Scopus, Science Direct, Cochrane were consulted, as well as the gray literature bases Britsh Library, OpenGrey.eu and Object View and Interaction Design (OVID). The following search strategy was used for the PUBMED database: (Otoacoustic emissions, spontaneous OR spontaneous otoacoustic emission OR spontaneous otoacoustic emissions) AND (auditory perceptual disorders OR auditory perceptual disorder OR auditory processing disorders OR disorder, auditory processing OR acoustic perceptual disorders OR auditory comprehension disorders).

Other search strategies are described in Appendix A.

No search filters were used, nor were there any limitations by year of publication or language.

The searches were carried out between the months of January and March 2021, an update was carried out in September 2023.

### Eligibility criteria

Only original articles of the cross-sectional type with a comparison group and that performed TEOAE suppression in individuals with and without CAPD were included, only studies carried out in the child population will be included. Studies with only comparative groups were selected considering that there are several ways to stimulate the efferent system through TEOAE suppression, making it necessary to compare populations with and without CAPD using the same stimulation parameters. Studies in non-human beings, studies in populations with hearing loss and with diagnosis of pathologies associated with CAPD were excluded.

### Selection of studies

The research was carried out by two independent reviewers (JDS and LCDH), in the absence of agreement, the study was evaluated by a third researcher (LGAV) for final decision-making.

The first phase of article selection was the reading of titles and abstracts, at the same time, of all identified studies. After excluding those that did not respond to the purpose of the study and did not meet the eligibility criteria, duplicate articles were excluded and then the remaining studies were read in full. Studies that did not meet the review proposal were excluded after this last step.

### Data extraction and quality assessment

The data extracted from the studies were: authors' names, year of publication, country, sample size, age range of the studied group, altered CAP tests, stimulation parameters for TEOAE suppression effect, analysis parameters performed to identify absence of contralateral TEOAE inhibition, TEOAE suppression results and differential tools used for CAPD screening.

To assess the quality of the studies and the risk of bias, the Newcastle Ottawa^14^ scale for cross-sectional studies was used.

The quality of scientific evidence was assessed using the Grades of Recommendation, Assessment, Development, and Evaluation (GRADE) system.^15^

### Statistical analysis

The outcomes of each study were divided into continuous variables (Mean ± SD). For continuous variables, the weighted difference of means was used to test the overall effect. The models of fixed effect (without heterogeneity) and random effect (with heterogeneity) were chosen using the inverse variance method (this measure of variability is directly related to the sample size, that is, the larger the sample size, the smaller the estimated variability and, consequently, the greater weight of the study in estimating the meta-analytical measure (hence the name inverse) and two-tailed Confidence Interval (95% CI) of 95%.

Higgins statistics (I^2^) was used to assess homogeneity between studies and low heterogeneity was considered if I^2^ < 50% and moderate and high heterogeneity if I^2^ ≥ 50%

Data were entered into an Excel spreadsheet and statistical analyzes were performed using the free software Revman 5.4 (http://r-project.org/, accessed on 09/20/2022), using the meta package.

## Results

**Figure 1:**
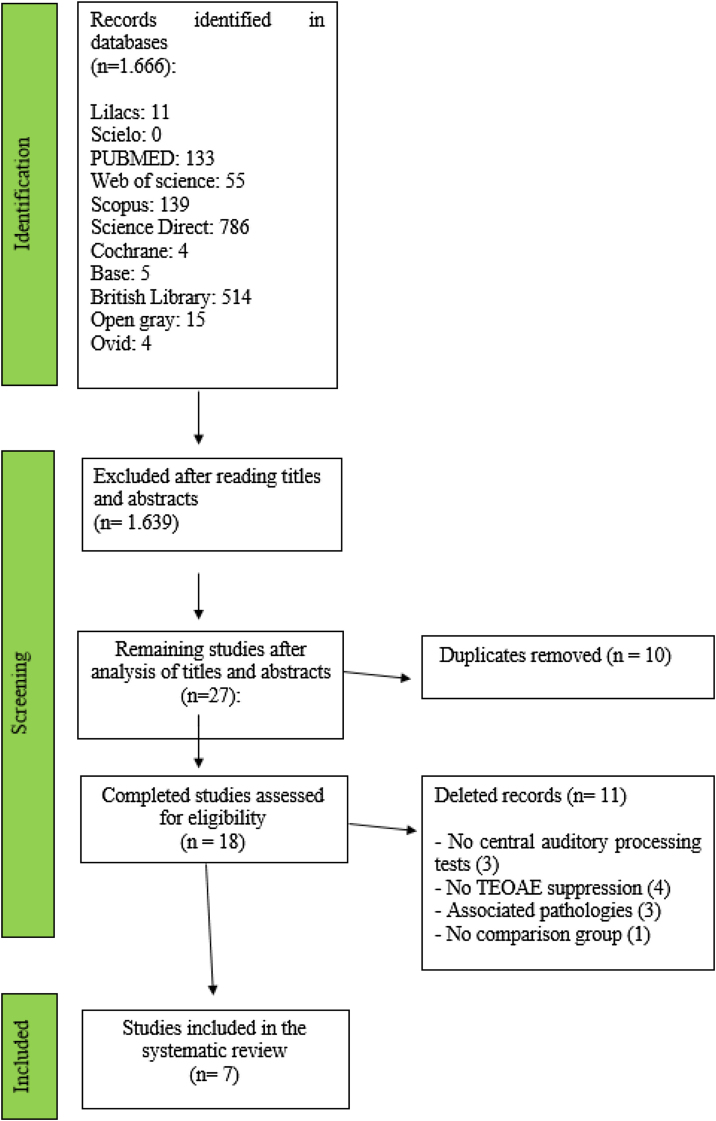
Article selection flow diagram.

In the initial search of the databases, 1666 titles were identified and, after reading the titles and abstracts, 27 studies remained. Of this amount, 10 appeared in duplicate and were removed, leaving 18 that were read in full. After reading the texts, 11 studies were excluded for the following reasons: (3) They included associated pathologies, (3) Absence of CAP tests, (4) Did not perform TEOAE suppression and (1) Absence of group without CAPD. At the end of the analysis process, seven studies were selected for this literature review, as described in Fig. 1.

Table 1 presents the data obtained from reading the included articles, according to the pre-established criteria for data extraction. All studies had as a population people who had CAPD without other associated conditions.

**Table 1:** Characteristics of the studies and population, stimulation parameters and main results.

Authors/Year	Age	Sample	CAP tests performed	OAE stimulation	Suppressing noise	MEMR	SNR	Suppression effect analysis	Presence of suppression effect	Supression values
DS Bezerra, RG Angrisani, LD Pereira, MF Azevedo, KZ Dias, 2021	7–14 years old	30	Sound Localization Test, Sequential Memory Test for Verbal Sounds, Sequential Memory Test for Nonverbal Sounds, White Noise Speech Test, Dichotic Digit Test, Random Interval Detection Test, and Duration Pattern Recognition Test with flute sounds.	ILO V6 equipment. Linear clicks with intensity between 75 and 85 db pe SPL and frequency spectrum of 2‒4 kHz, stimulation with a total of 260 sweeps.	Contralateral stimulation with broadband noise of 60 dB SPL, emitted by the second channel of the OAE equipment. Both ears were tested.	AR activation above 70 dB, OAET stimulus intensity 65 dB, no other controls.	3 dB	Subtraction of amplitude values with noise from the amplitude obtained without noise.	Amplitude reduction by at least 0.5 dB SPL after addition of contralateral noise	SG: RE: 0.55/LE: 0.41 CG: RE: 0.71/LE: 0.87
Smart, JL, Kuruvilla-Mathew, A., Kelly, AS e Purdy, SC, 2019	7–12 years old	63	Frequency Pattern Test, Dichotic Digits Test, Compressed and Reverberated Words, Gaps In Noise, 500-Hz tone in white noise, s0 n0 versus sp n0 masking level difference, Speech in Spatial Noise.	SmartTrOAE (Intelligent Hearing Systems) equipment. Linear clicks at 60 dB peSPL, and frequency spectrum of 1–4 kHz. Window of 30 ms.	Contralateral stimulation with white noise of 65 dB SPL, generated by a audiometer and presented through insert headphones. Both ears were tested	AR activation above 80 dB, OAET stimulus intensity 60 dB no other controls.	6 dB	Calculated in two ways: Absolute suppression - Subtraction of amplitude values with noise from the amplitude obtained without noise. Normalized suppression - Responses were transformed into linear scales, the amplitude with noise (linear) was normalized to amplitude without noise (baseline) and applied as a percentage change from the baseline amplitude.	Presence of the suppression effect considered when the values were positive	CG: 0.9/SG: 1.6
TS Mattsson, O Lind, T Follestad, K Grøndahl, Wilson, S Nordgård, 2019	8–14 years old	43	Filtered Words, Digit Dichotic, SCAN-C Battery Competitive Words Test, Frequency Pattern Test; Gap in Noise, Binaural masking differential threshold.	ILO V6 equipment. Clicks at 60 ± 3 dB pe NPs, frequency spectrum between 1–4 kHz and 50 clicks/s using linear differential stimulus paradigm. Presentation of 1040 stimuli without noise and 1040 stimuli with noise, separated by blocks of 260 sweeps. 2–20 ms window.	Contralateral stimulation with white noise with a spectrum of 0.2–20 Khz, generated by the OAE equipment, at 60 dB SPL. Both ears were tested.	AR activation above 70 dB, OAET stimulus intensity 60 dB, no other controls.	3 dB	Calculated in two ways: Absolute suppression - Subtraction of amplitude values with noise from the amplitude obtained without noise. Normalized suppression - Responses were transformed into linear scales, the amplitude with noise (linear) was normalized to amplitude without noise (baseline) and applied as a percentage change from the baseline amplitude.	x	Absolute suppression (dB SPL): SG: RE: 1.1/LE: 1.0
										CG: RE: 1.0/LE: 1.3
										Normalized suppression (%): SG: RE: 11.3/LE: 10.6
										CG: RE: 10.3/LE: 13.0
T Morlet, K Nagao, LA Greenwood, RM Cardinale, RG Gaffney, T Riegner/2019	7–12 year age	19	SCAN-3 Test for Children, Bamford-Kowal-Bench Sentences in Noise, Dichotic Digit Test, Frequency Pattern Test, Overlapping Spondaic Word Test, Random Gap Detection Test, Phonemic Synthesis Test, and Auditory Continuous Performance Test.	SmartTrOAE (Intelligent Hearing Systems) equipment. Linear clicks at 65 dB peSPL. Presentation of blocks of 500 stimuli. Window of 8–18 ms.	Ipsilateral, contralateral and binaural stimulation with broadband noise with a spectrum from 0 to 16 KHz at 65 dB SPL. Three OAE recordings were made, randomly interspersed with different noise conditions, with an inter-stimulus interval of 10 ms. Both ears were tested.	No controls.	6 dB	Absolute suppression - Subtraction of amplitude values with noise from the amplitude obtained without noise.	x	Contralateral noise: CG: RE: 2.9/LE: 2.0
										SG: RE: 1.9/LE: 2.7
										Ipsilateral noise: CG: RE: 4.2/LE: 3.0
										SG: RE: 3.1/LE: 2.7
										Binaural noise: 65 dB: CG: RE: 47./LE: 5.0
										SG: RE: 5.2/LE: 5.3/50 dB: CG: RE: 2.8/3.0
										SG: RE: 3.6/LE: 4.2/40 dB: CG: RE: 2.2/LE: 2.0
										SG: RE: 2.3/LE: 3.4/30 dB: CG: RE: 0.9/OE: −0.1
										SG: RE: 1.6/LE: 2.5
FAR Burguetti. RMM Carvallo/2008	9–10 years old	88	Speech with noise and dichotic with alternating disyllables.	ILO 92 equipment, Otodynamics version 5.61. Click stimuli, collection of alternating responses with and without noise every 20 stimuli, generating 200 sweeps of linear stimuli. 20 ms window.	Contralateral stimulation with white noise, generated by channel B of the OAE equipment, at 60 dB HL. Both ears were tested.	No controls.	5 dB	The sum of the values obtained in the absence of noise were subtracted from the sum of the values obtained with contralateral noise.	Presence of the suppression effect considered when the values were positive.	CG: 1.50
										SG: 1.26
SGG Sanches RM Carvallo/2006	7–11 years old	51	CG: Dichotic Disyllable Listening Test, Speech in Noise Test for monosyllabic words.	ILO 92 Otodynamic Analyzer equipment, version 5.61. Linear clicks adjusted to reach a maximum pressure of 60 dB and non-linear clicks with a pressure of 60 dB and can be increased every 5 dB to reach a maximum of 80 dB. Presentation of blocks of 260 stimuli. 2.5–20 ms window	Contralateral stimulation with white noise at 60 dBSPL, emitted by the second channel of the OAE equipment. Both ears were tested.	No controls.	ND	Absolute suppression - Subtraction of amplitude values with noise from the amplitude obtained without noise.	They were considered present when the result of the subtraction resulted in positive values.	Linear clicks: CG: 1.79
			SG: Disyllable Dichotic Listening Test, Pediatric Speech Intelligibility, Nonverbal Dichotic Test, Speech in Noise Test, Sound Localization Test, and Verbal and Nonverbal Sequential Memory Test.							SG 1: 1.11
										SG 2: 1.25
										Non-linear clicks: CG: 1.90
										SG 1: 1.04
										SG 2: 1.39
C Muchnik, DA Rotha, R Othman-Jebara, H Putter-Katz, EL Shabtaia, M Hildesheimer, 2004	8–13 years old	30	Competitive sentences test, Speech in noise test, Differential masking threshold, Gap detection.	ILO88 OAE analyzer equipment (Otodynamics Ltd, v. 4.2). Clicks with a frequency spectrum of 1–4 kHz, produced by rectangular electrical pulses of 80 μs and 50 clicks/s. Stimulation with a total of 260 sweeps. With a window of 2.5–20.48 ms during stimulation (standard window) and subsequently applied a window of 8.0–20.48 ms (later time).	Contralateral stimulation with white noise of 40 dB SPL, generated by a Beltone B200c audiometer and presented through insert headphones. Both ears were tested.	AR activation above 80 dB, no other controls.	3 dB	Pearson correlation was performed to assess the reliability of Q1 and Q2 recordings. Then, the conditions of Q1 and n, in addition to Q2 and N, were compared and repeated measures analysis of variance (ANOVA) was applied, followed by an average value of the suppressions for each subject.	Two cutoff points were defined to categorize reduced suppression values: 0.6 and 1.0 dB.	Default window: SG: LE: 1.05/RE: 0.89
										CG: LE: 1.61/RE:1.57
										Later time window: SG: LE: 1.20/RE: 1.62
										CG: LE: 2.72/RE: 2.68

SG, Study Group; CG, Comparative Group; RE, Right Ear; LE, Left Ear; dB, Decibel; HL, Hearing Level; SPL, Sound Pressure; SPL, Sound Pressure Level; kHz, Kilo-Hertz; OAE, Otoacoustic Emissions; ms, Milliseconds; AR, Acoustic Reflex.

For the definition of the groups, comparative and study, the studies used a battery composed of validated tests, some of them with translation into Brazilian Portuguese and others available only in the language of the countries of origin and/or with translation into other languages. The criteria used for inclusion in the groups were heterogeneous between the studies, in five of them the presence of alterations in at least two tests was required.^4^, ^16^, ^17^, ^18^, ^19^, ^20^ Four studies showed which tests had altered results for the study population.^4^, ^16^, ^19^, ^20^

In addition to the battery of evaluation tests, the presence of behavioral signs or complaints of changes in the CAP^19^, ^21^ and the report of professionals in the area to define the diagnosis and the repercussions on the development of daily activities^17^ were also considered.

As for the stimuli used for TEOAE, only the click type was mentioned with a linear and non-linear paradigm, however the presentation intensity varied between 60 and 85 dB. The signal-to-noise ratio accepted by studies for OAE analysis varied between 3^17^, ^19^, ^20^ and 6 dB.^16^, ^18^

Regarding the suppressor stimulus used, it was mostly presented contralaterally,^4^, ^16^, ^17^, ^19^, ^20^, ^21^ using white noise^4^, ^17^, ^19^, ^21^ or broadband,^16^, ^18^, ^20^ spectrum variation between 0–20 kHz^18^, ^19^ and intensity between 40–65 dB. The suppressor noise was generated by the OAE equipment or by the audiometer.

The analysis of the suppression effect was performed in an absolute manner and one of the studies performed clinical normalization of the data. The included studies considered the activation of the efferent system with the observation of reduced TEOAE amplitude after adding the contralateral noise, two studies evaluated the suppression of TEOAE as present or absent, considered present when the subtraction of the values obtained originated positive numerical results.^4^, ^21^

Study quality was assessed according to the Newcastle-Ottawa Scale adapted for cross-sectional studies, shown in Table 2. All studies were classified as good. As for the representativeness of the sample, despite not having sample calculation, the studies were considered unrepresentative of the target population. Only one of the studies did not score due to the selection of participants, as the comparative group was selected from schools and did not perform auditory processing tests to exclude the possibility of central alterations.^17^

**Table 2:** Assessment of the risk of bias of the included studies. Subtitle: The categories analyzed in the Newcastle-Otawa Scale evaluate studies regarding the risk of bias. The lower the number of stars received, the greater the risk of bias. The selection category can score a maximum of five stars, the comparability category can score up to two stars and the results category can score a maximum score of three stars. Studies that score up to four points are considered unsatisfactory, five to six points are satisfactory and above seven points are good studies.

Authors/Year	Selection				Comparability	Results	
	Sample representativeness	Sample size	Non-respondent	Risk factors	Controlled confounding factors	Result evaluation	Statistical test
Burguetti 2008	B*	B	C	A**	B**	B**	A*
Mattsson 2019	C	B	A*	A**	B**	B**	A*
Muchnik 2004	B*	B	C	A**	B**	B**	A*
Sanches 2006	B*	B	C	A**	B**	B**	A*
Morlet 2019	B*	B	C	A**	B**	B**	A*
Smart 2019	B*	B	A*	A**	B**	B*	A*
Bezerra 2021	B*	B	C	A**	B*	B**	A*

The same study scored positively in the category of non-respondents, considering that explained the loss of participants during data collection and obtained a satisfactory response rate.^17^ As for the control of confounding factors, one of the studies scored negatively for not presenting homogeneity of the data regarding the genders of the participants, the study group being composed of 14 male and 1 female individual and the comparative group with 8 males and 7 females.

**Figure 2:**
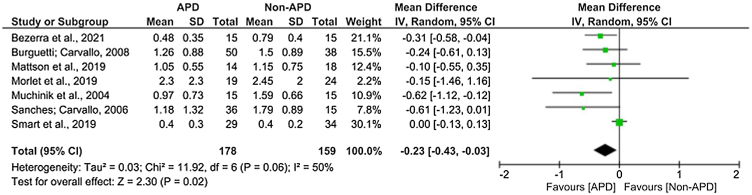
Forest Plot of the presence of the OAE suppression effect of the studies included in the meta-analysis. APD, Auditory Processing Disorder; IV, Inverse Variance; CI, Confidence Interval; SD, Standard Deviation.

In Fig. 2, the difference between the experimental and comparative groups was statistically signiﬁcant (*p* = 0.02), both when considering the fixed or random effects model, favoring the experimental group. Moderate heterogeneity (I^2^ = 50%) and significance with *p* = 0.06 were verified, that is, the data from the studies are homogeneous. The overall mean value of the differences was −0.23 (−0.43 to −0.03). The studies by Muchinik^19^ and Sanches^21^ showed greater differences between the experimental and comparative groups, and the study by Morlet et al.^18^ showed the greatest variability with a larger confidence interval (−1.46 to 1.16). Only the study by Bezerra^20^ and Muchinik^19^ has, individually, statistically significant differences between the APD and Non-APD groups.

Table 3 shows the evaluation of the quality of the scientific evidence with the application of the GRADE^15^ method in analyzing the quality of scientific evidence for the outcome reduction in OAE amplitude after the addition of competitive noise. It was possible to analyze only one outcome for this systematic review, reduction in TEOAE amplitude after adding competitive noise in individuals with CAPD. During the analysis, there was a 2 reduction of one level due to indirect evidence, since the question guiding the review requires greater generalization of the data regarding age group, however the included studies mostly evaluated children and adolescents, only one of them evaluated the adult population and the elderly individuals did not They have been observed. Just as there was a downgrade of one level due to imprecision, the minimum size of information was not reached for quantitative outcomes. At the end of the evaluation, a very low level of evidence was obtained for the analyzed outcome.

**Table 3:** Application of the GRADE method for analyzing the quality of scientific evidence for the outcome reduction in OAE amplitude after the addition of competitive noise.

Certainty assessment							Nº of participants		Effects		Certainty	Importance
Studies	Study design	Risk of bias	Inconsistency	Indirect evidence	Inaccuracy	Other considerations	APD	Non-APD	Relative (95% CI)	Absolue (95% CI)		
**Menor amplitude das supressões das EOA em indivíduos com TPAC**												
7	Observational studies	Not serius	Not serious	Serious	Serious	None	159	137	‒	Mean 0.38 less (0.58 less to 019 less)	⊕⊖⊖⊖	IMPORTANT

CI, Confidence Interval.

## Discussion

TEOAE suppression is still not a widespread assessment tool in clinical practice, despite its easy implementation. The functionality of the test to identify possible changes in the auditory efferent system has still been investigated by scientific research in the area of hearing.

This review aimed to investigate, through a systematic review of the literature, whether there are differences in the results of TEOAE suppression tests in individuals with and without CAPD.

For the inclusion of participants in the research, CAP tests were performed, and the criteria for participation were heterogeneous between studies. Five of them used as a rule for inclusion in the study group to present at least two altered tests in the battery used,^4^, ^16^, ^17^, ^18^ while two others evaluated the scores in the tests performed individually, being necessary to present alterations in only one test associated with the presence of signs behavioral or complaints of CAP alterations.^16^, ^20^, ^21^

Currently, it is known that the results of the MOC evaluation can be affected by the activation of the Middle Ear Muscle (MEMR). However, precautions taken, such as the verification of acoustic reflex thresholds, the frequency range stimulated in the examination, and the intensity of the stimulus used for the OAE, assist in reducing the possibilities of MEMR interference in the findings.^17^, ^18^ Only four of the analyzed studies used criteria to minimize the participation of this muscle in the examination results.^16^, ^17^, ^19^, ^20^

All studies performed absolute subtraction for the analysis of the suppression effect, with the amplitude value obtained in noise subtracted from the amplitude value obtained in silence. Different analyses were carried out to define the absence and presence of EOAT suppression. One of the studies conducted two different analyses, one through absolute subtraction and another through normalized subtraction. This study did not point out a criterion for verifying the presence or absence of suppression, it only evaluated which group presented higher amplitudes of the effect. Both analyses presented the same response patterns, the study pointed out the absence of MOC alterations related to CAPD.^17^

Another article also used an analysis based on two response acquisitions, with the aim of verifying the stability and reliability of the EOA suppression responses. After verifying, through statistical tests, the low variability of the values, the authors performed an average of the two results obtained, resulting in the suppression value of the EOA for each subject.^19^

Only four studies defined criteria for the presence of EOA suppression effect,^4^, ^19^, ^20^, ^21^ all pointed out as being the obtaining of positive values after the previously mentioned subtraction. Two of them presented cut-off points for the verification of the effect, which brings greater reliability in the findings and allows the reproduction of the method and analysis.^19^, ^20^

After analysis of the findings, it was observed that although individuals with CAPD have lower TEOAE suppression values, the results are not statistically significant in relation to the comparative group. Only three studies pointed out that responses to suppression were significantly lower in participants with CAPD.^20^, ^21^ However, both studies used low SNR, a factor that compromises the quality of the findings.

One of theses studies verified lower mean values and greater numbers of absences of the TEOAE suppression effect in the study group when the values were obtained with the use of linear clicks and justifies this finding by saying that the linear click cancels part of the response that would be influenced by linear artifacts, which allows for greater clarity regarding the findings.^21^

Despite the lack of statistical significance, the lower TEOAE suppression values suggest that in individuals with CAPD there is a lower inhibitory effect, which shows less activation of the MOC in these participants.^4^, ^21^ This finding may point to neurophysiological evidence of reduced function of MOC in this population.^19^, ^20^

Only one research carried out more than one form of stimulation (ipsilateral, contralateral and bilateral), despite finding no differences between the groups analyzed, it was observed that both groups presented higher suppression values in the suppression condition with binaural noise.^18^ This finding can be justified because binaural stimulation activates the crossed and uncrossed fibers of the MOCB, which provides a better visualization of the system's function. It is believed that in contralateral stimulation only a third of the MOCB fibers are stimulated, which reflects the evaluation of only a small portion of the system.^22^

Furthermore, it was observed that children with CAPD had less prominent suppression in the Left Ear (LE) when binaural noise was presented at intensities between 30 dB SPL and 65 dB SPL, compared to those without hearing complaints. Such an effect can be obtained, probably because individuals with CAPD have less activation of the Medial Olivocochlear System (MOC) on the left.^18^

One of the studies modified the acquisition window and verified that the time window after sending the stimulus showed higher TEOAE suppression values in both groups, that is, excluding the first eight milliseconds from the analysis. The justification for this fact is that this decision reduces the influence of the external and middle ears on the suppression findings.^17^

As for the ear advantage, the findings are quite divergent and some studies indicate that there were greater responses in the Right Ear (RE) in both evaluated groups.^14^, ^17^ One of the studies found that the study group had a greater advantage for the LE, while the comparison group such an effect was verified in RE.^10^ Muchinik and collaborators^17^ showed that individuals with CAPD had higher values for RE, justified by a reduced force of the olivocochlear system effect in the left ear of this group.

However, other studies did not significantly observe such an effect.^10^, ^15^, ^16^ The lack of ear effect in TEOAE suppression can be justified by the age of the investigated population, since asymmetries can develop throughout childhood, therefore, they may not have been identified in the individuals observed because they were adults.^14^

Differential tools such as questionnaires were also used for CAPD screening, the Amsterdam Inventory for Auditory Disability and Handicap (AIADH) can be used to identify listening complaints in individuals with or without CAPD, thus listing hearing difficulties in situations of silence or noise competitive.^13^ In addition, behavioral signs can be analyzed based on the reports of professionals in the area to define the diagnosis and the repercussions on the development of daily activities.^14^

The studies indicate divergent opinions when it comes to the feasibility of the technique in patients with CAPD. Matsson et al.^14^ point out that the use of the TEOAE suppression test is not feasible in this population, considering that the CAPD population did not show different responses to the TEOAE suppression when compared to the comparative group.

On the other hand, Muchinik,^20^ Bezerra,^21^ Sanches^22^ and their collaborators. support the use of this procedure as a complementary tool to the audiological diagnosis for patients with CAPD. These studies indicate the presence of OAE suppression when the amplitude values with noise addition are lower than the amplitude obtained in silence, with these results observed in the study groups. Thus, individuals with CAPD would show results with negative values in OAE suppression. as it can identify alterations in the function of the MOC in the processing of sound information.

The meta-analysis carried out for the present review indicated that the average TEOAE suppression amplitudes obtained in the studies favor the experimental group, thus, in addition to presenting homogeneity in terms of the results obtained, it also points out that there are less robust results efferent responses from the auditory pathways in the CAPD group. Despite this result, it is important to note that only two studies did not surpass the nullity line, which indicates that none of the other studies obtained significant statistical differences between participants with and without CAPD.

It is also important to highlight that most of these studies did not control important factors for obtaining OAE suppression responses, such as MEMR measurement, low SNR and the application of only contralateral noise. Therefore, it is possible to point out the inclusion of articles with low methodological quality as a limitation of the current study. This finding points to the need to carry out new original studies that control these confounding factors in MOCB responses.

Thus, adding to the diagnostic battery of behavioral, electroacoustic and electrophysiological tests that indicate altered auditory skills. In addition, they point out that a can be used to monitor the results of interventions based on auditory training.

## Conclusion

Despite obtaining a quantitative analysis that indicates the presence of a significant effect estimate for the CAPD group, most of the articles individually do not indicate the presence of statistically significant differences between the study and comparison groups.

The current study identified a wide range of stimulation parameters and analyses performed in the OAE suppression examination. This highlights the need for a protocol that standardizes the measurement of OAE suppression and its findings, aiming to reduce the inclusion of results unrelated to the olivocochlear system. Following this standardization, it will be possible to conduct new studies to explore the relationship between the OAE suppression test and the presence of central hearing difficulties across different age groups.

## Funding

This work was carried out with the support of the Coordination for the Improvement of Higher Education Personnel ‒ Brazil (CAPES) ‒ funding code 001.

## Conflicts of interest

The authors declare no conflicts of interest.
